# Supplementary material for: Content-rich biological network constructed by mining PubMed abstracts
Source: BMC Bioinformatics. 2004 Oct 8;5:147. doi: 10.1186/1471-2105-5-147 (PMC528731; doi:10.1186/1471-2105-5-147)
Supplement: Additional File 2 — The original results of the above study (non-essential files are deleted to keep the file size under the limit set by BMC bioinformatics). [file 1471-2105-5-147-S2.bz2 › chilibotAdditionalFile2/dip05/242ID8921390E798/html/RAD52_UBE2I.html]

 


 **RAD52** and **UBE2I** 
  
Found 3 abstracts in PubMed, retrieved 3.  
 

 What does Google say? 
 PDF only 
| .edu only 

---

**Interactive relationship** (e.g. stimulation, inhibition, etc)

**Neutral relationship**- The interaction of  **UBE2I**  with  **RAD52**  is mediated by  **RAD52**  s self association region.  Ref: 8921390 Genomics, 1996
- Using the human  **RAD52**  protein as the bait in a yeast two hybrid system, we have identified a human homolog of yeast UBC9  [ **UBE2I** ] , designated  **UBE2I** , that interacts with  **RAD52** , RAD51, p53, and a ubiquitin like protein UBL1.  Ref: 8921390 Genomics, 1996

**Non-interactive relationship** (e.g. studied together, co-existance, homology, etc.)

- Associations of  **UBE2I**  with  **RAD52** , UBL1, p53, and RAD51 proteins in a yeast two hybrid system.  Ref: 8921390 Genomics, 1996
- These results suggest that the  **RAD52**  dependent processes, cell cycle control, p53 mediated pathway s, and ubiquitination interact through human  **UBE2I** .  Ref: 8921390 Genomics, 1996
